# Reciprocal expression of INSM1 and YAP1 defines subgroups in small cell lung cancer

**DOI:** 10.18632/oncotarget.20572

**Published:** 2017-08-28

**Authors:** Karen McColl, Gary Wildey, Nneha Sakre, Mary Beth Lipka, Mohadese Behtaj, Adam Kresak, Yanwen Chen, Michael Yang, Vamsidhar Velcheti, Pingfu Fu, Afshin Dowlati

**Affiliations:** ^1^ Division of Hematology and Oncology, Case Western Reserve University, Cleveland, OH, USA; ^2^ Department of Pathology, University Hospitals Cleveland Medical Center, Cleveland, OH, USA; ^3^ Case Comprehensive Cancer Center, Case Western Reserve University, Cleveland, OH, USA; ^4^ Department of Population and Quantitative Health Sciences, Case Western Reserve University, Cleveland, OH, USA; ^5^ Division of Hematology and Oncology, Cleveland Clinic Foundation, Cleveland, OH, USA; ^6^ Division of Hematology and Oncology, Case Western Reserve University, University Hospitals Seidman Cancer Center, Cleveland, OH, USA

**Keywords:** INSM1, YAP1, small cell, RB1, CDK4/6

## Abstract

The majority of small cell lung cancer (SCLC) patients demonstrate initial chemo-sensitivity, whereas a distinct subgroup of SCLC patients, termed chemo-refractory, do not respond to treatment. There is little understanding of how to distinguish these patients prior to disease treatment. Here we used gene expression profiling to stratify SCLC into subgroups and characterized a molecular phenotype that may identify, in part, chemo-refractive SCLC patients. Two subgroups of SCLC were identified in both cell lines and tumors by the reciprocal expression of two genes; *INSM1*, a neuroendocrine transcription factor, and *YAP1*, a key mediator of the Hippo pathway. The great majority of tumors expressed INSM1, which was prognostic for increased progression-free survival and associated with chemo-sensitivity in cell lines. YAP1 is expressed in a minority of SCLC tumors and was shown in cell lines to be downstream of the retinoblastoma protein (RB1) and associated with decreased drug sensitivity. RB1 expression in SCLC cell lines sensitizes them to CDK4/6 inhibitors. Wild-type *RB1* mutation status, used as a surrogate marker of YAP1 expression, was prognostic for decreased patient survival and increased chemo-refractory tumor response. Thus, the reciprocal expression of INSM1 and YAP1 appears to stratify SCLC into distinct subgroups and may be useful, along with *RB1* mutation status, to identify chemo-refractory SCLC patients.

## INTRODUCTION

Small cell lung cancer (SCLC) represents about 15% of all lung carcinomas [1]. SCLC is distinguished from non-small cell lung cancer (NSCLC) by its neurosecretory phenotype, which includes dense core secretory granule formation and neuropeptide secretion. The expression of multiple neuroendocrine genes; including synaptophysin, chromogranin A, and CD56 (NCAM1), are often used in the diagnosis of SCLC [2, 3].

From a clinical standpoint, SCLC has been viewed as a homogeneous disease characterized by rapid growth and metastasis. SCLC is remarkable for its initial chemo-sensitivity followed by early relapse and resistance to further therapy [4, 5]. Physicians have long recognized a distinct subgroup of SCLC patients, however, with so-called primary refractory disease that demonstrate an absence of tumor shrinkage in response to initial chemotherapy. Chemo-refractive behavior in SCLC tumors is a particularly dire prognosis because the second-line drugs used for SCLC provide only limited benefit to patient survival.

Here, we describe two subgroups of SCLC based upon the reciprocal expression of two genes; *INSM1*, a neuroendocrine gene, and *YAP1*, a key mediator of the Hippo pathway. Importantly, the expression of these two genes may predict chemo-response in SCLC tumors. Furthermore, chemo-refractory tumors may contain functional retinoblastoma (RB1) protein, making them susceptible to drugs targeting CDK4/6.

## RESULTS

### INSM1 and YAP1 expression define two subgroups of SCLC cells

**Figure 1:**
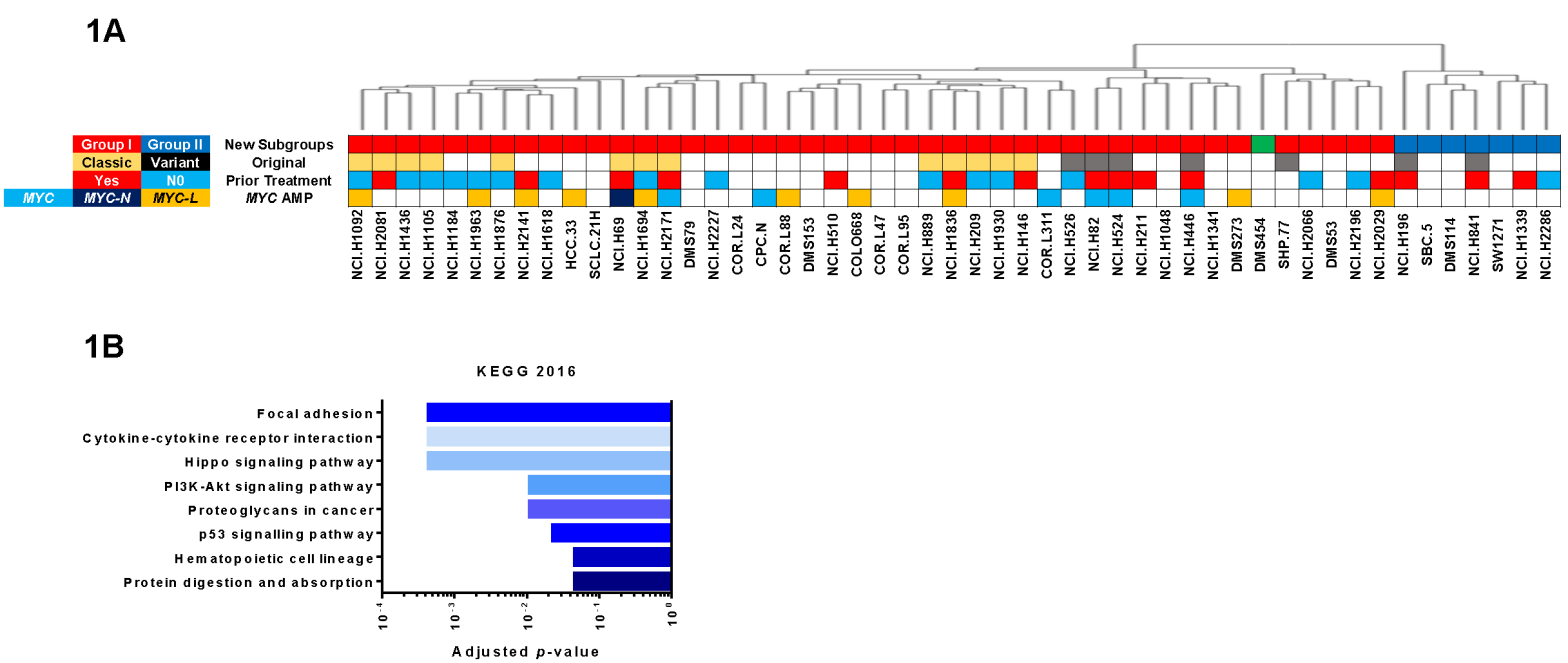
Identification of subgroups in SCLC cell lines **A.** Dendrogram at top reproduced from reference 6. Colored bars on top indicate whether cells are members of Group I (red), Group II (blue) or Group III (green). Original classification of cells by Gadzar and Minna given below, when available [7]. Prior treatment with drug or radiation extracted from reference [36]. *MYC* amplification determined from COSMIC database. Blank (white) boxes indicate no value found. **B.** Results of Enrichr analysis of 132 significant genes that were increased 1.5-fold in Group II cell lines.

Previously [6] we performed unsupervised gene expression clustering analysis on 51 SCLC cell lines in the Cancer Cell Line Encyclopedia (CCLE) and identified two subgroups based upon ∼4500 ‘significant’ genes (*p* < 0.05) (Supplementary Figure S1 and Supplementary Table S1); we refer to these as Group I (red bars in Figure 1A) and Group II (blue bars) cells. A third subgroup consisted of only a single cell line (DMS454, green bar). Our subgroups did not align with the ‘classic *vs* variant’ designations originally proposed by Gazdar and Minna for SCLC cell lines [7], although our clustering clearly segregated them. There was also no apparent correlation of our clustering with prior therapeutic treatment of the original tumor. Interestingly, no *MYC*-family amplifications were present in Group II cells. The *INSM1* gene, which encodes a neuroendocrine transcription factor [8], showed the highest relative expression in Group I *vs* II cells. Remarkably, three Hippo pathway genes were among the most highly expressed genes in Group II cells; *YAP1* encodes a central mediator in the Hippo signaling pathway and *CTGF* and *CYR61* are downstream transcriptional targets [9]. Bioinformatic analysis revealed that Group I genes did not significantly map to any KEGG pathways whereas Group II genes were significantly associated with many KEGG signaling pathways, including the Hippo pathway (Figure 1B, Supplementary Table S2). We focused on exploring *INSM1* and *YAP1* expression as biomarkers for Group I *vs* II cells, respectively.

**Figure 2:**
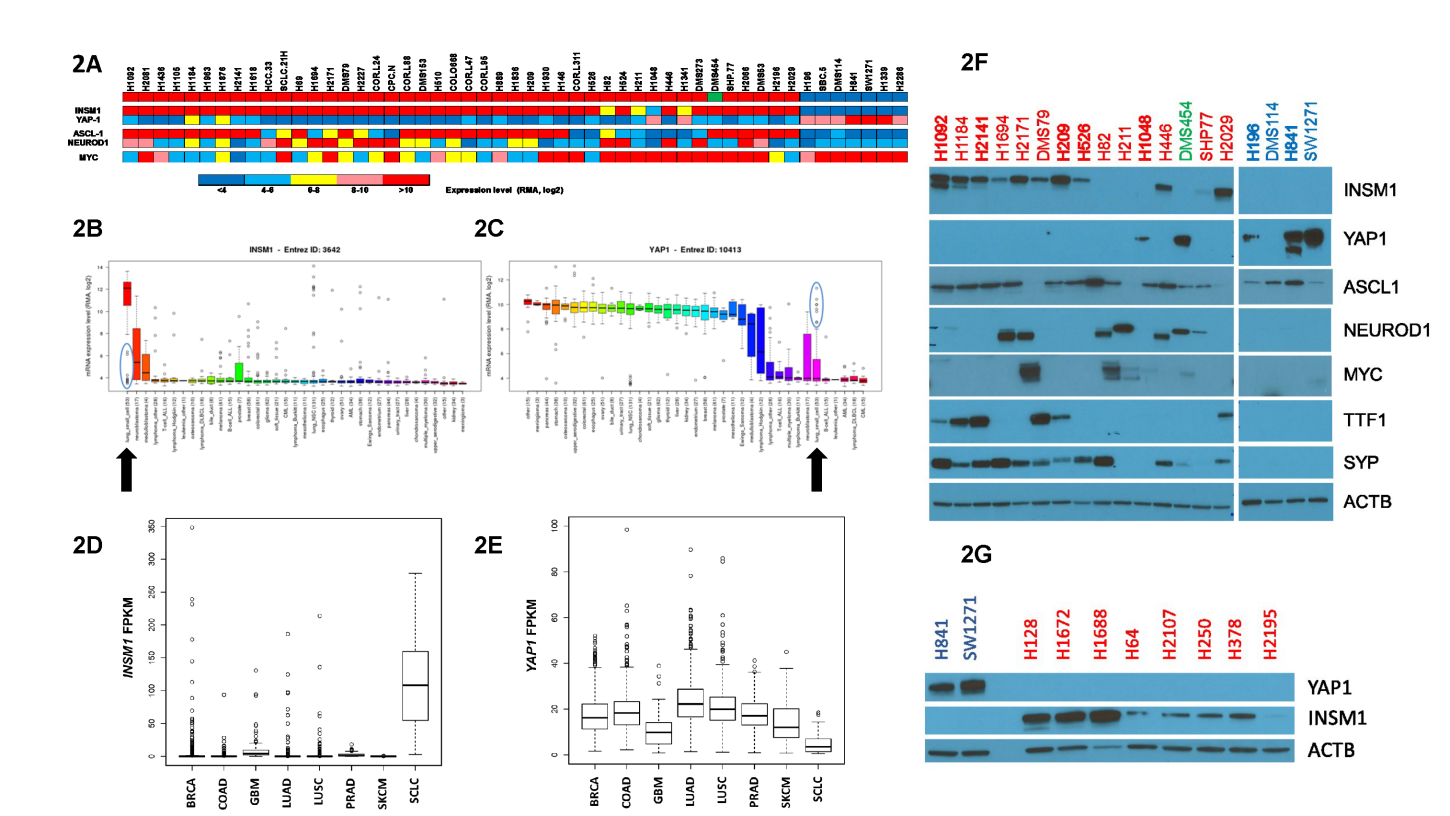
*INSM1* and *YAP1* expression in SCLC cell lines **A.** Expression of various genes in individual SCLC cell lines. Cell lines are arranged, left to right, following their gene expression clustering assignments, as shown in Figure 1A and Supplementary Figure S1. Gene mRNA expression for individual genes, listed on left, was color-coded based upon the RMA, log2 values, obtained from the CCLE. Comparison of *INSM1* **B.** and *YAP1* **C.** mRNA expression levels among different types of cancer cell lines in the CCLE. The line within individual boxes represents the median expression value of all cells tested and the individual circles represent ‘outlier’ cells whose expression values do not fall within the 25-75% quantile of values measured for that gene (represented by the box). The blue circles highlight the subgroup of outliers present in SCLC cells. Arrows highlight SCLC. **D.** Boxplot comparing *INSM1* expression among tumors using RNAseq data from the TCGA and Rudin et al [14]. Tumor types listed on x-axis with *INSM1* mRNA level listed as FPKM on the y-axis. Boxplots similar to panels B and C except use FPKM expression values. In addition to SCLC, other cancers analyzed were breast (BRCA), colorectal adenocarcinoma (COAD), brain glioblastoma (GBM), lung adenocarcinoma (LUAD), lung squamous cell carcinoma (LUSC), prostate (PRAD) and skin (SKCM). **E.** Same as panel D except for *YAP1* mRNA expression. **F.** Western blots of protein lysates. Targeted protein listed on right. Cells are arranged on blot, left to right, in identical order as shown on clustering diagram in Figure 1A. Cell names are written in colored text to designate their subgroup classification. Group II cells were run on a separate gel but otherwise analyzed on the same day and conditions as Group I cells. β-actin (ACTB) was used as a loading control. **G.** Same as panel F except for SCLC cell lines not included in the CCLE dataset.

When the mRNA expression levels of *INSM1* and *YAP1* in individual cell lines were plotted following the clustering analysis, they showed reciprocal expression of these two genes in Group I *vs* Group II cells (Figure 2A). Interestingly, the expression of two genes suggested as potential classifiers of SCLC subgroups, *ASCL1* and *NEUROD1* [10-12], each demonstrated low expression in Group II cells and variable expression among Group I cells (Figure 2A). *MYC* expression has also been recently suggested as a potential classifier of SCLC, in particular of a variant phenotype [13]. Interestingly, the distribution of high *MYC*-expressing cells by our clustering was significantly enriched in Group II *vs* Group I cells (*p* = 0.042). We next looked at the relative expression of *INSM1* and *YAP1* among all cancer cell lines in the CCLE. SCLC demonstrated the highest *INSM1* expression and among the lowest *YAP1* expression (Figures 2B, 2C). This analysis also clearly identified a subgroup of SCLC cells (circled) for each of these two genes that were outliers to an otherwise uniform level of expression; these outliers were largely Group II cells. When we compared the mRNA expression levels of these two genes in tumor tissues using RNAseq data from TCGA, we again found that *INSM1* expression was highest in SCLC, whereas *YAP1* expression was among the lowest (Figures 2D, 2E).

Western blotting experiments confirmed that *INSM1* and *YAP1* were differentially expressed at a protein level; INSM1 was present in most Group I cells whereas YAP1 was present in most Group II cells (Figure 2F). Importantly, INSM1 and YAP1 expression were mutually exclusive in 15 SCLC cell lines; the remaining four SCLC cells lines expressed little or no detectable INSM1 and YAP1 protein. To confirm this, another eight SCLC cell lines not included in the CCLE analysis were western blotted and seven expressed only INSM1 while one (H2195) expressed neither protein (Figure 2G). Examination of other neuroendocrine genes showed that ASCL1 expression demonstrates near uniform expression across all SCLC cell lines, in contrast with its mRNA expression, whereas NEUROD1 expression was restricted to Group I cells (Figure 2F). MYC expression was apparent only in *MYC*-amplified cell lines. When the expression of two proteins used clinically in the diagnosis of SCLC was examined, thyroid transcription factor 1 (TTF1 of *NKX2-1* gene) expression was, interestingly, reciprocal to NEUROD1 expression in Group I cells while synaptophysin (SYP) expression appeared to parallel that of INSM1 (Figure 2F). Thus, protein expression studies validated the reciprocal expression of INSM1 and YAP1 in SCLC cell lines.

### INSM1 and YAP1 expression can stratify SCLC tumors

**Figure 3:**
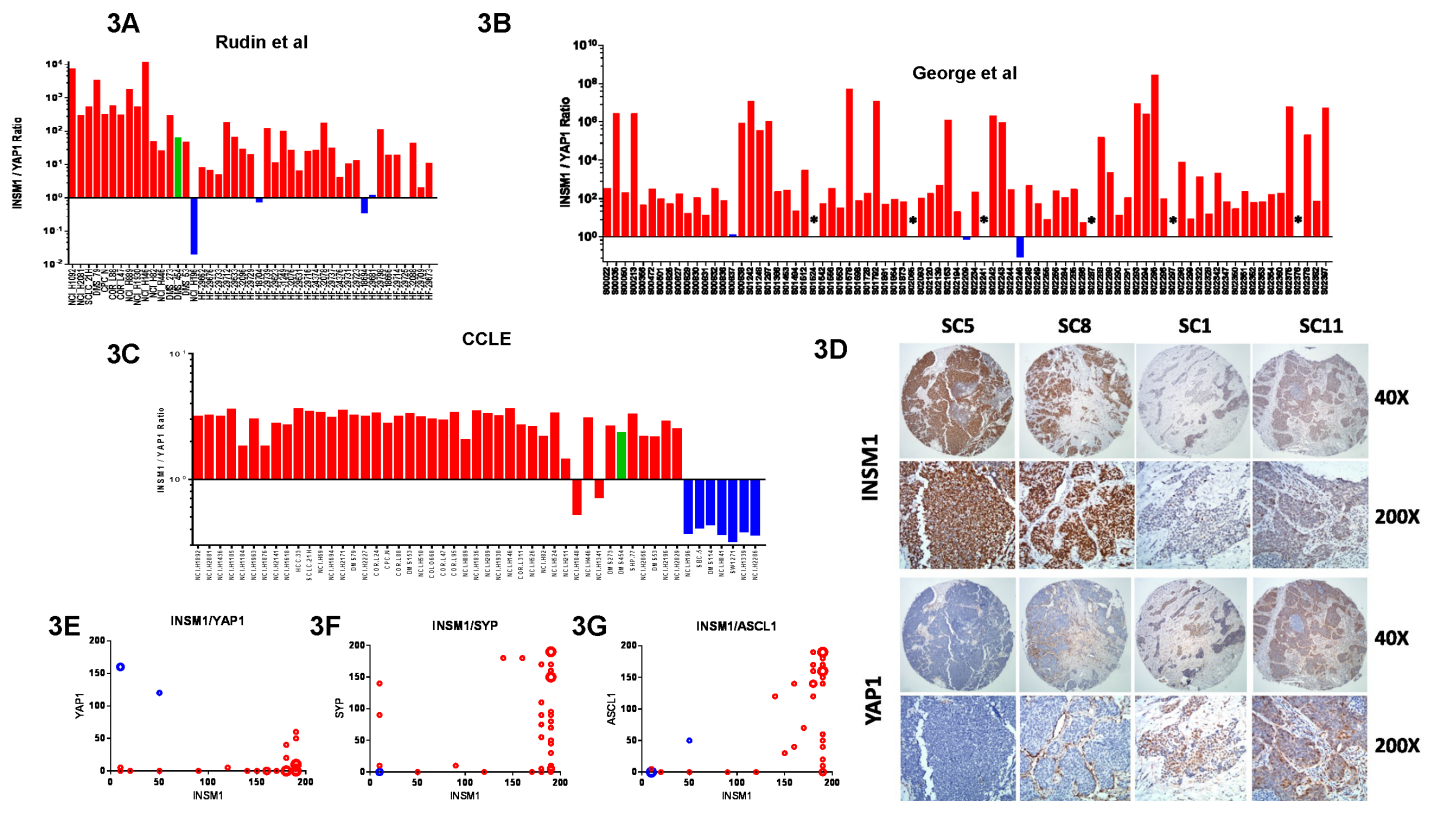
Identification of subgroups in SCLC tumors *INSM1*/*YAP1* mRNA expression ratios using data from the **A.** Rudin et al [14], **B.** George et al [15], and **C.** CCLE datasets [33]. Colored bars represent potential subgroup assignments in **A.** and **B.**. *Tumors with *YAP1* mRNA = 0 in **B.** thus ratio cannot be calculated. **D.** INSM1 and YAP1 IHC on consecutive sections of four distinct tumor cores in discovery TMA, identified at top, each shown at two magnifications (shown to right). Slides were counterstained to show histology. Plots of INSM1 *versus* YAP1 **E.**, SYP **F.** and ASCL1 **G.** IHC scores in discovery TMA using data from Supplementary Table S3. Size of circles proportional to number of cores represented. Blue circles represent cores with a high YAP1 score.

To determine if subgroups of human SCLC tumors could be identified based upon *INSM1/YAP1* mRNA expression, we initially examined RNAseq data from Rudin et al [14] and gene array data from George et al [15] and found that both datasets contained tumors with low *INSM1/YAP1* mRNA expression ratios (Figure 3A and 3B, respectively), as observed for Group II cell lines (Figure 3C), albeit less frequently.

We next looked for differential protein expression of INSM1 and YAP1 in SCLC tumors by IHC using a discovery TMA containing 22 patient tumor specimens. Strong INSM1 staining was detected in the majority of tumors (Figure 3D, see cores SC5 and SC8), whereas YAP1 staining was positive in only a few tumors (see cores SC1 and SC11). Both stains were predominantly nuclear. The pattern of INSM1 and YAP1 staining supported the idea of reciprocal expression of these two proteins in tumors (Figure 3E, see Supplementary Table S3 for full IHC scoring results). Furthermore, when the TMA was stained for two other neuroendocrine genes, SYP and ASCL1, there did not seem to be any strong correlation with INSM1 staining (Figures 3F, 3G), indicating that INSM1 may represent a novel neuroendocrine biomarker in SCLC.

### SCLC subgroups predict drug response

**Figure 4:**
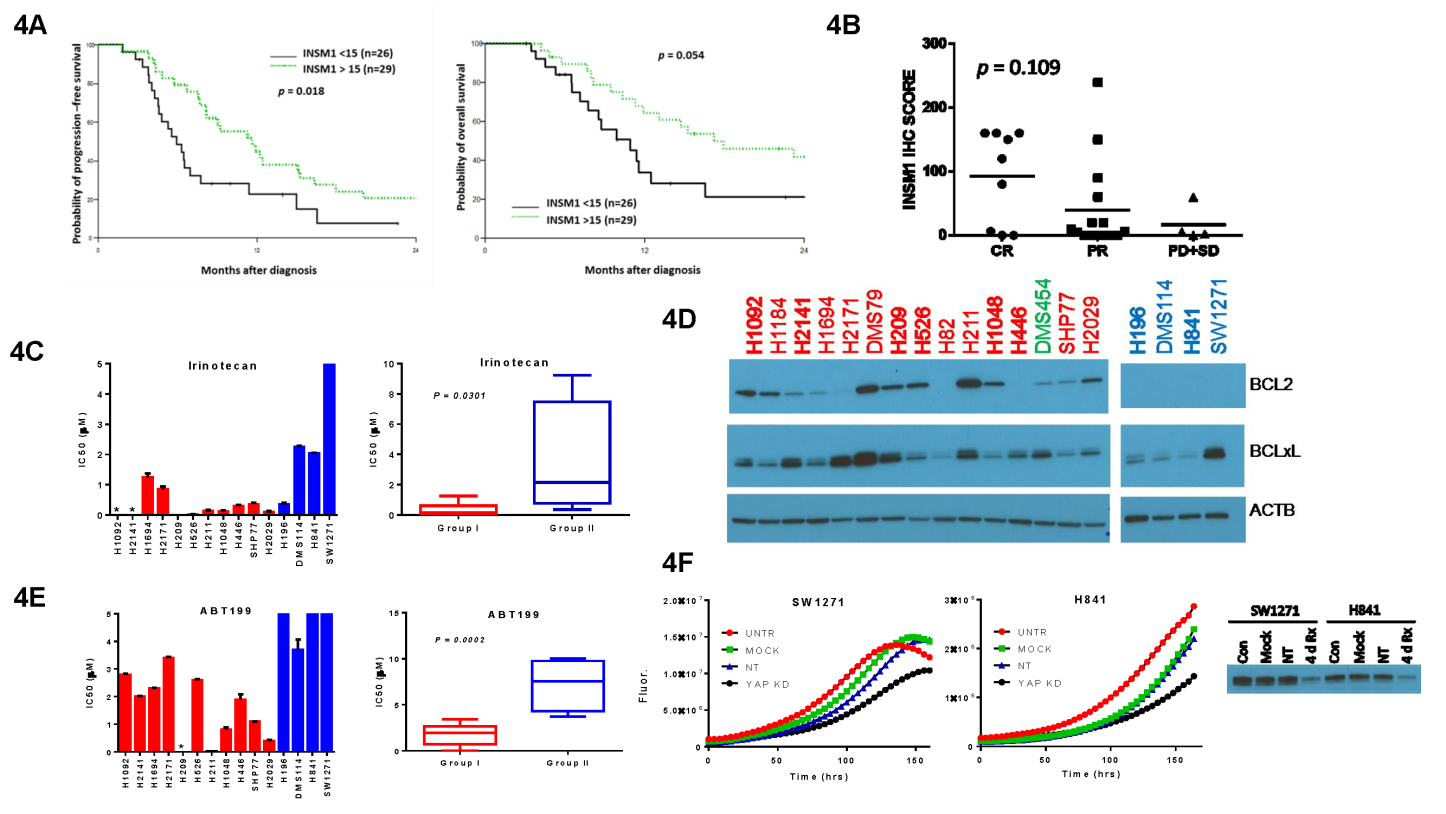
Chemo-sensitivity of SCLC subgroups **A.** Survival analysis based upon INSM1 IHC scores from the second TMA. Restricted to those patients with OS ≥ 3 mo. The median INSM1 expression score was 15 (range: 0 - 240) and the mean was 51 (STD: 70.9). **B.** Correlation of INSM1 IHC score with chemo-response (*N* = 28) in the second TMA cohort. Response was graded as complete response (CR), partial response (PR), progressive disease (PD) or stable disease (SD) using RECIST criteria. Mean IHC values are shown by horizontal bars. **C.** IC50 values for irinotecan inhibition of cell growth listed for individual cell lines (left) as well as for cell line subgroups (right). Individual cell line IC50 values represent mean ± SEM of 1-2 independent experiments and are shown as either red (Group I) or blue (Group II) bars depending on their subgroup assignment. Cells are arranged on x-axis, left to right, in identical order to the clustering diagram in Figure 1A. *Cell line not tested. Boxplots represent mean ± SEM of individual IC50 values for a Group with p values showing significance between Groups. **D.** Western blot of cell lysates shown as in Figure 2F but probed for BCL2 and BCLxL. **E.** Same as in panel C except for ABT199. **F.** Growth curves for SW1271 and H841 cells after knockdown by 500 nM YAP1 siRNA (KD) or non-targeting siRNA (NT) compared to untreated (CON) or mock-transfected cells. Bottom shows western blot validating YAP1 knockdown after 4 days of treatment.

To determine if the expression of INSM1 or YAP1 had any clinical significance, we stained a second TMA containing 55 additional SCLC tumor samples for just INSM1 expression because its robust staining and frequent positivity made it easier to score than YAP1. Our analysis showed that higher INSM1 expression was associated with a trend toward greater overall survival (OS) (*p* = 0.054) and significantly increased progression-free survival (PFS) (*p* = 0.018) (Figure 4A). These survival differences may be explained by a trend toward increased chemo-response with increased INSM1 IHC staining (*p* = 0.109) (Figure 4B).

To directly investigate if Group I cells with high INSM1 expression are more chemo-sensitive, we measured the drug sensitivity of our two subgroups of SCLC cell lines to three standard agents used in the treatment of SCLC; cisplatin, etoposide and irinotecan. Group I cells were significantly more sensitive to irinotecan than Group II cells (Figure 4C). This was not due to differential expression of topoisomerases (Supplementary Figure S2) or previous exposure to therapy (Figure 1A). Cisplatin and etoposide also showed a trend toward increased efficacy in Group I cells (Supplementary Figure S3).

*BCL2* has received much attention as an actionable, pro-survival gene that is highly expressed in the majority of SCLC [16], so we hypothesized that it may be selectively expressed in Group I cells. Indeed, our clustering analysis showed *BCL2* mRNA to be statistically higher in Group I cells (*p* = 0.0069) (Supplementary Figure S4) and subsequent western blots validated BCL2 protein expression only in Group I cells, which was not observed for another pro-survival family member, BCLxL (Figure 4D). Selective expression of BCL2 sensitized Group I cells to the BCL2-specific inhibitor ABT199 (Figure 4E), supporting the idea that our subgroups predict response to multiple drugs.

We attempted to identify drugs that selectively target Group II cells. Unfortunately verteporfin, a recently identified drug that inhibits Hippo pathway signaling by preventing YAP1 interaction with its transcriptional partner TEAD1 [17], was not selective in our hands. As proof-of-principal, however, we could demonstrate that knockdown of YAP1 inhibited cell growth in both H841 and SW1271 cells (Figure 4F), indicating that other small-molecule inhibitors targeting the Hippo pathway might be effective against Group II cells. Other drugs we tested that showed no difference in efficacy between Group I and II cells included selumetinib, mubritinib, 17-AAG, and AUY922.

### RB1 regulates YAP1 expression and confers sensitivity to CDK inhibitors

**Figure 5:**
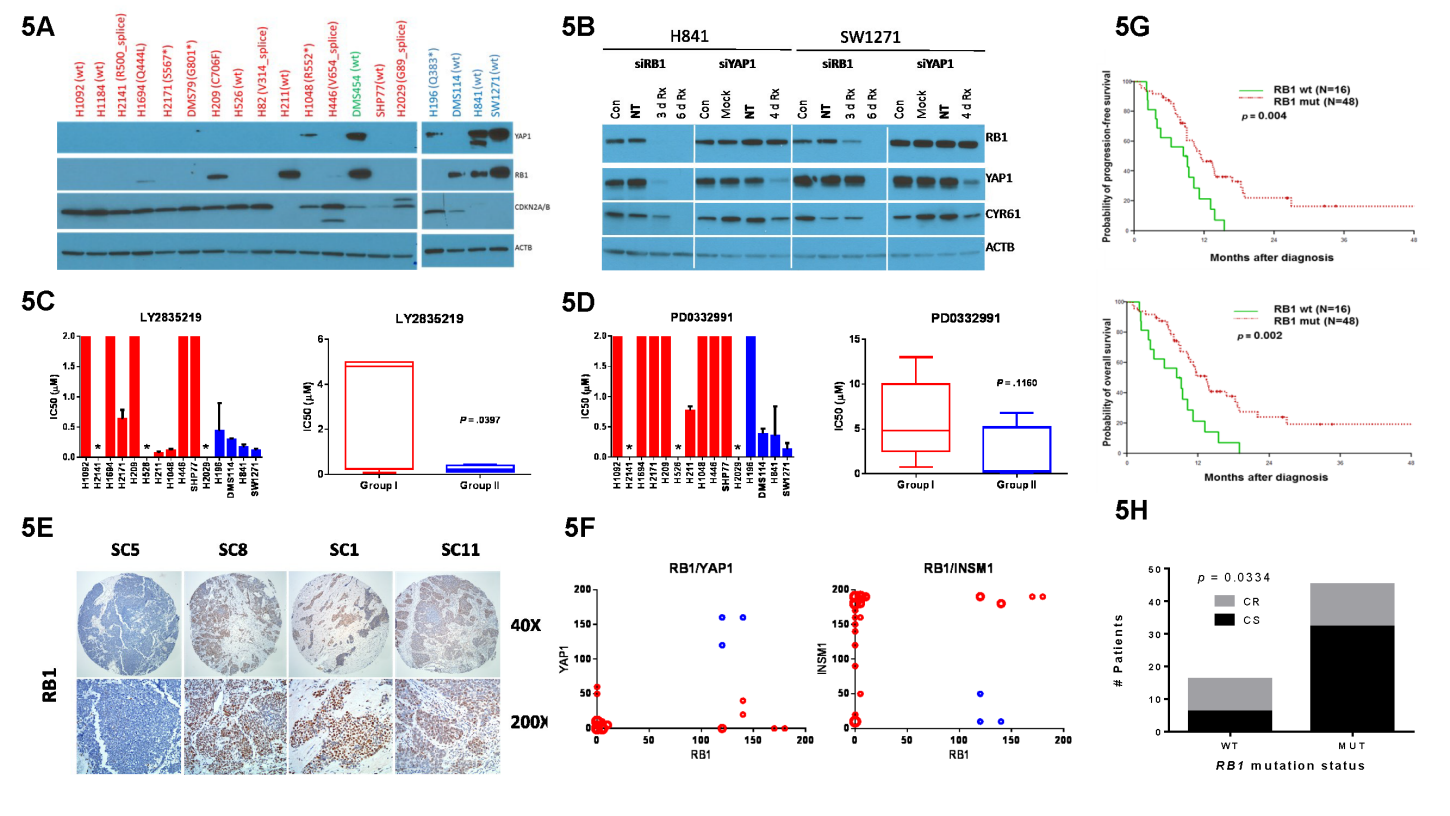
Role of RB1 in YAP1 expression and SCLC subgroup stratification **A.** Western blots of protein lysates shown as in Figure 2F. Targeted protein listed on right. YAP1 blot is reproduced from Figure 2F for easy comparison. RB1 exon mutation status obtained from COSMIC shown in parentheses after cell line name. **B.** Western blot results of transient knockdown of RB1 or YAP1 in SW1271 and H841. Cells were treated with gene-specific siRNA (Rx) or non-targeting siRNA (NT) and results compared to untreated (Con) or mock-transfected (Mock) cells. Protein lysates were prepared after the indicated numbers of days. IC50 values for CDK4/6 inhibitors LY2835219 **C.** and PD0332991 **D.** on individual cell lines. Individual cell line IC50 values are represented as in 4C. Bars with no error bars have IC50 values > 2 µM. **E.** RB1 IHC on consecutive sections of same four tumor cores as shown in Figure 3D, each shown at two magnifications (shown to right). Slides were counterstained to show histology. **F.** Plots of RB1 *versus* YAP1 and INSM1 IHC scores using data from Supplementary Table S3. Size of circles proportional to number of cores represented. Blue circles represent cores with a high YAP1 score. **G.** Survival analysis based upon *RB1* mutation status in the genomic cohort (*N* = 64). A binary (+/-) scoring system was used for mutation status and did not consider the type of mutation or the number of *RB1* mutations per tumor. **H.** Correlation of *RB1* mutation status with initial chemo-response in genomic cohort of panel G (*N* = 61). Response was graded as chemo-sensitive (CS) or chemo-refractory (CR) based upon the standard RECIST criteria for SCLC.

Because Group II cells represented a small subgroup of all SCLC cell lines, we were curious to determine their relationship to a small subgroup of SCLC cell lines we identified in a previous study which expressed retinoblastoma protein (RB1) [18]. *RB1* mRNA was enriched in Group II cells in our clustering analysis (*p* = 0.0415) (Supplementary Figure S4) and we found by western blotting that RB1 was co-expressed with YAP1 in three cell lines (DMS454, H841, SW1271) (Figure 5A). These three cell lines also demonstrated the highest YAP1 expression levels. In contrast, the remaining YAP1-positive cell lines, H1048 and H196, did not co-express RB1 and also demonstrated much lower YAP1 protein expression. RB1 expression was mostly associated with a wild-type (wt) exon mutation status and its functionality in YAP1-positive cell lines was suggested by decreased or absent CDKN2A/2B expression, previously shown as an alternate mechanism to disable the RB1 pathway in lung cancer [19]. We used siRNA knockdown experiments to directly address the relationship of RB1 and YAP1 in SCLC (Figure 5B). RB1 knockdown in both H841 and SW1271 cells led to a loss of YAP1 expression, whereas YAP1 knockdown had no effect on RB1 expression, placing RB1 upstream of YAP1. Either RB1 or YAP1 knockdown decreased expression of the Hippo downstream target CYR61. Taken together, these results indicate that a subgroup of SCLC cell lines have a functional RB1 signaling pathway leading to YAP1 expression and Hippo pathway activation.

We then hypothesized that if YAP1 expression is functionally linked to RB1, Group II cells should be sensitive to CDK4/6 inhibitors such as LY2835219 and PD0332991. Indeed, these two drugs demonstrated increased efficacy toward Group II cells (Figure 5C, 5D), with H211 and H196 cells being notable exceptions explainable by their RB1 expression status.

To validate that YAP1 and RB1 are co-expressed in SCLC tumors as well, we stained our discovery TMA for RB1. Although we obtained only 9 positives in 45 cores (Figure 5E and Supplementary Table S3), strong YAP1 expression was only observed in cores with strong RB1 expression (compare cores SC1 and SC11 in Figures 3D and 5E). However, 6 of 9 cores with strong RB1 staining demonstrated little to no YAP1 expression (see core SC8) and the vast majority of tumor cores were negative for RB1 and YAP1 staining (Figure 5F), which mirrors our observations in SCLC cell lines.

Finally, based upon our previous results with INSM1 (Figure 4A), we hypothesized that YAP1 expression should have an opposite, or negative effect on survival. We used wild-type (wt) *RB1* mutation status as a surrogate, albeit imperfect marker of YAP1 expression (see cell line data in Figure 5A) and examined its association with survival in a third cohort of SCLC patients with tumor exon-sequencing data (Supplementary Table S4) [18]. We found that wt *RB1* mutation status was present in a minority (∼25%) of all SCLC patients in this cohort but was associated with significantly shorter overall (*p* = 0.002) and progression-free (*p* = 0.004) survival compared to patients with mutant RB1 (Figure 5G). Decreased survival in patients with wt RB1 was associated with significantly increased chemo-refractory tumor response relative to patients harboring mutant *RB1* tumors (*p* = 0.0364) (Figure 5H). Interestingly, the only two patients with *CCND1* amplification in the genomic cohort both displayed wt *RB1* mutation status, and one of these two patients also harbored the only *CDKN2A/B* loss detected. Taken together, our results suggest that decreased survival in YAP1/RB1-positive patients may result from an increased percentage of chemo-refractory patients.

## DISCUSSION

Compared to other cancers, particularly breast and NSCLC, the use of ‘omics’ to identify subgroups in SCLC is in its infancy. Several whole exome sequencing (WES) studies and one whole genome sequencing (WGS) study on SCLC [14, 15, 20-22] are beginning to provide insight into the biology of this disease. Subgroup stratification in SCLC is hindered, however, by the wide heterogeneity and low frequency ( < 10%) of most genes mutated in SCLC beyond the two which define this cancer, *TP53* and *RB1*. The repeated failure of past attempts to find effective targeted therapies for SCLC is likely explained by this mutational heterogeneity and reinforces our need to identify subgroups of SCLC patients that have appropriate biomarkers with clinical relevance.

Here, we propose a stratification of SCLC into two subgroups based on the expression of two genes, *INSM1* and *YAP1*. Our working model, borrowing the terminology of Gazdar and Minna, is that INSM1 positive tumors represent the ‘classic’ every-day SCLC that is chemo-sensitive, whereas INSM1 negative tumors, which include YAP1 positive tumors, represents a ‘variant’ form of SCLC that is chemo-refractive. The vast majority of SCLC cell lines and tumors express high INSM1 mRNA and/or protein, consistent with the high proportion of chemo-sensitive SCLC patients. Evidence that INSM1 expression predicts a chemo-sensitive drug response is based upon the increased efficacy of irinotecan in Group I cell lines (Figure 4C) and a trend toward chemo-responsiveness with increased INSM1 IHC scores in tumors (Figure 4B). By contrast, only a small proportion of SCLC patients are chemo-refractory, about 10-20%, in agreement with the small size of our YAP1 positive/INSM1 negative subgroup in cell lines or tumors. Beyond the cell line data mentioned above, other evidence that YAP1 expression predicts a chemo-refractory drug response comes from SCLC patients harboring wt RB1 (Figure 5H). Although we use this data as a surrogate for YAP1 expression, we feel it is reasonable based on our cell line (Figures 5A, 5B) and IHC data (Figure 5E). Thus, the strength of our proposed model is based upon many intersecting lines of evidence, involving both cell lines and multiple patient cohorts. This approach is necessitated by the well-known difficulty in obtaining single large, annotated cohorts of SCLC tumor tissue for analysis. Our model is supported by a recent study showing that YAP1 loss occurs in the majority of SCLC and is associated with increased chemo-sensitivity in a different cohort of SCLC cell lines [23]. In addition, YAP1 expression is associated with increased chemo-resistance in ovarian and NSCLC cancer [24, 25]. Unfortunately, the mechanisms underlying how YAP1 increases chemo-resistance or, conversely, INSM1 increases chemo-sensitivity, remains unknown.

**Figure 6:**
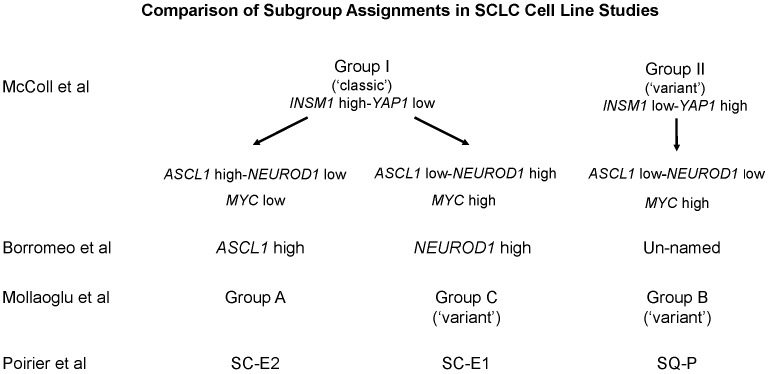
Comparison of SCLC subgroups among multiple studies Subgroup assignments and/or gene expression data of cell lines using Figure 1A of this study, Figure 1A of Borromeo et al [12], Figure 3A of Mollaoglu et al [13], and Figure 5A of Poirier et al [11] were compared and vertically grouped by their general similarity to one another. The group names used by each individual study are given to facilitate comparisons.

Recent studies have focused on *ASCL1* and *NEUROD1* mRNA expression as important classifiers of SCLC [11-13]. They all identify three subgroups in SCLC: *ASCL1*^high^/*NEUROD*1^low^, *ASCL1*^low^/*NEUROD1*^high^ and *ASCL1*^low^/*NEUROD1*^low^. We can find parallels to these three subgroups in our clustering analysis if we focus only on mRNA expression data (Figure 6). Our Group II clearly equals the *ASCL1*^low^/*NEUROD1*^low^ subgroup. Our Group I subgroup includes both the *ASCL1*^high^/*NEUROD1*^low^ and *ASCL1*^low^/*NEUROD1*^high^ subgroups because *ASCL1* and *NEUROD1* largely demonstrate reciprocal mRNA expression in Group I cells, although they are not spatially segregated. If specific cell lines are examined, considerable overlap of subgroups among all of these studies can be found; particularly if Group I cells with high *MYC* mRNA expression are segregated out. At a protein level, however, ASCL1 fails to distinguish any subgroups, while NEUROD1 is restricted to Group I cells (Figure 2F). The consistently low levels of MYC protein expression are also uninformative for stratification, except for those cells with *MYC* amplification, as reported previously [26]. Taken together, a general consensus can be found at a gene mRNA expression level that can segregate SCLC into subgroups, however, this stratification becomes less clear at a protein expression level, which does not always parallel mRNA expression.

The recent characterization of INSM1 DNA binding sites as super-enhancers in SCLC [27] is consistent with the idea that INSM1 may control global gene expression programs producing a neuroendocrine phenotype in the majority of SCLC. While this is a role increasingly attributed to ASCL1 [10, 12], our results demonstrate near uniform expression of ASCL1 among most SCLC cell lines, including Group II cells, and we also found ASCL1 to be more variably expressed in our SCLC TMA compared to INSM1. INSM1 has been shown to be an upstream regulator of ASCL1 expression in two SCLC cell lines [28], thus, the relative importance of these two SCLC transcription factors in regulating the neuroendocrine phenotype needs further clarification.

INSM1 expression represents an attractive biomarker because its intense IHC staining is easily translated to the clinical lab and is compatible with the small amounts of biopsy tissue typically obtained from SCLC tumors. INSM1 expression might also represent a novel biomarker in SCLC, as it did not correlate with either SYP or TTF1 expression (Figures 2F, 3F). While it was recently reported that a CNV signature in circulating tumor cells may also predict chemo-response in SCLC [29], the wide application of this exciting finding may be limited by the advanced technology required.

Finally, our finding that YAP1 expression is downstream of RB1 signaling may provide a missing mechanistic link in generating the ‘classic’ SCLC phenotype as RB1 expression is lost in the majority of SCLC and also during acquired resistance to erlotinib in NSCLC to SCLC transformation [30]. The apparent functional signaling of RB1 in some SCLC cell lines with wt *RB1* mutation status suggests that CDK4/6 inhibitors should be tested for efficacy against similar SCLC tumors.

## MATERIALS AND METHODS

### Cell lines

All cell lines were purchased within the last three years from ATCC, except for DMS454 (Sigma), and have not been authenticated. Cells were maintained as recommended by the supplier. To generate RFP stable cell lines, CellPlayer NucLight Red lentivirus was purchased (Essen Bioscience) and transduced into wild type cells using an MOI of 3. After 48 h, 0.5 µg/ml puromycin was added for selection.

### Western blotting

Protein lysates (40 µg) were analyzed as described previously [31] using 4-20% Criterion gels (Bio-Rad). Antibodies were purchased from: Cell Signaling (YAP1 #4912, BCLxL #2764, SYP #12270, Topoisomerase I #12286, TTF-1 #12373, RB1 #9309, CYR61 #14479, CDKN2A/B #4824, c-MYC #5605, NEUROD1 #4373), Santa Cruz (INSM1 sc-271408, BCL2 sc-7382), LifeSpan Bio (ASCL1 #C177728), Pharmingen (Topoisomerase II, #556597) and Sigma (Actin, A-5441).

### Drug treatment

5,000 RFP transduced cells were treated with 1nM, 10nM, 100nM, 1µM and 10µM of drug (all from Selleck) in duplicate or triplicate and allowed to grow for up to 136 h in the IncuCyte Zoom (Essen Bioscience) with fluorescent scans taken every 4 h. Growth curves were generated showing total RFP integrated intensity over time and 3-5 time points were chosen during log phase growth to calculate the average growth inhibition per drug dose. IC50 values for each experiment were determined by linear regression analysis (average % inhibition *vs* log dose) using PRISM software. The IC50 value shown for individual cell lines is the average of 1-2 independent experiments ± SEM. The IC50 values for individual cell lines were used to calculate the Group means ± SEM and compared (I *vs* II) by unpaired two-tailed *t*-tests using PRISM software (Figures 4C, 4E, 5C, 5D). The same set of cell lines was routinely used for IC50 measurements, except where noted.

### siRNA knockdown of YAP1 and RB1

Knockdown of YAP1 was accomplished with ON-TARGETplus siRNA smartpool (Dharmacon #L-012200-00-0005, 500 nM) for human *YAP1* or Non-Target siRNA. SW1271-RFP and H841-RFP cells were electroporated at 160 Volts and 500µF capacitance using a 2mm cuvette. Cell growth was followed in the IncuCyte Zoom while the majority of cells were taken for western blot analysis after 96 h. Growth curves were generated using total RFP integrated intensity. Knockdown experiments were repeated at least 2 independent times.

Knockdown of *RB1* was accomplished with RB1 siRNA, sc-29468 (Santa Cruz Biotechnology). Ten microliters of 10 µM RB1 siRNA mixed with 100 µl of media and 3.0 µl of transfection reagent were added to 100 µL of siRNA Transfection Medium (Santa Cruz Biotechnology), kept at room temperature for 20 min and then added drop-wise to six well plates. After incubation for 6 h at 37°C, the media was replaced with fresh complete media and the incubation continued. After 48 h the cells were harvested, plated and were re-transfected following the same protocol. After another 48 h, the cells were harvested and total RNA or protein was extracted for analysis.

### Immunohistochemistry

Immunohistochemistry (IHC) was performed as described previously [32] except for SYP, which was performed by the hospital clinical lab. Tissue micro arrays (TMAs) were incubated with the following dilutions of primary antibodies: 1:400 INSM1, 1:200 YAP1, 1:100 ASCL1, and 1:1600 dilution RB1. The antibodies used were identical to those used in western blotting. Staining was graded on a scale from 0-2+ or 0-3+, depending on the specific cohort, antibody and stain intensity, and focused on nuclear staining for INSM1, YAP1 and RB1. The final IHC score was calculated as the (staining intensity) x (percentage IHC positive tumor cells), yielding a final scoring range of 0 to 200-300. Scoring was performed by a thoracic pathologist (MY) who was blinded to the outcome data.

### Tissue microarrays

The discovery TMA (*N* = 22 patient tumors) was assembled by the Case Comprehensive Cancer Center Tissue Resources core from formalin-fixed, paraffin-embedded (FFPE) tissue blocks. At least two 1 mm cores were taken from each patient tumor, with the majority of specimens collected from 2012-2014. A second TMA (*N* = 55 patient tumors) was obtained from Dr. Vamsidhar Velcheti. The second TMA contained single cores of each patient tumor, with the majority of specimens collected from 2007-2012. Construction and analysis of each TMA had prior approval from their respective local IRBs. Patient tumor specimens were randomly chosen for inclusion in TMAs based upon availability of archival tissue. Key descriptive statistics for each cohort are given in Supplementary Table S5. Chemo-responses in patient cohorts were based upon RECIST criteria.

### Genomic cohort

This cohort is an extension of that used in reference 18. The patients included in this cohort are sequential patients that presented to our institution who had tumor biopsies analyzed by targeted exome sequencing as part of their standard care. All patient biopsies were taken pre-treatment. The diagnosis of SCLC was made by a thoracic pathologist. For statistical outcome analyses, the specific type or number of *RB1* mutations per tumor were not considered, only its presence/absence. Chemo-response of the genomic cohort was based upon RECIST criteria and was determined for all patients. Supplementary Table S4 lists the RB1 mutations and chemo-response for individual patients while Supplementary Table S5 provides summary descriptive statistics for this cohort. This ongoing retrospective genomic analysis is approved by our local IRB.

### Dataset analyses

The publically available Cancer Cell Line Encyclopedia (CCLE) gene expression data was downloaded from http://broadinstitute.org/ccle [33] and calculated as described in reference 6 to obtain Figures 1A, 2A-2C, 3C and Supplementary Figure S1 and Supplementary Table S1. The RNAseq data obtained in the study by Rudin et al [14] was downloaded from the European Genome database and analyzed as in reference 6 to obtain Figures 2D, 2E and 3A. Briefly, Tophat [34] was used to do the alignment. Then Cufflinks [35] was employed to obtain the FPKM (fragments per kilobase per million) values. The transcriptome files of the other cancers (breast: BRCA, colorectal adenocarcinoma: COAD, brain glioblastoma: GBM, lung adenocarcinoma: LUAD, lung squamous cell carcinoma: LUSC, prostate: PRAD and skin: SKCM) were downloaded from the TCGA data portal (https://gdc.cancer.gov/). The FPKM values were extracted from those files. The boxplots (Figures 2D, 2E) were generated by R, version 3.2.2. The gene expression array data of George et al [15] was obtained from Supplementary Table S1, and the *INSM1* and the *YAP1* values (NM_001130145) were used in calculations for Figure 3B. The association of Group I and Group II significant genes with KEGG pathways was determined using the Enrichr bioinformatic tool (http://amp.pharm.mssm.edu/Enrichr/) using only those genes that demonstrated 1.5-fold enrichment in each subgroup (150 Group I and 132 Group II genes).

### Statistical analysis

Overall survival was measured from the date of diagnosis to the date of death and censored at the date of last follow-up for survivors. Progression-free survival was measured from the date of diagnosis to the date of disease progression or the date of death, whichever occurred first, and censored at the date of last follow-up for survivors without disease progression. Survivor distribution was estimated using Kaplan-Meier methods and differences in survival between groups was examined by the log-rank test (Figures 4A, 5G). Fisher’s exact test was used to examine the association of two categorical factors (Figures 2A, 5H) and one-way ANOVA was used to determine the difference of continuous measures among three groups (Figure 4B). Heatmap along with unsupervised hierarchical clustering was used for gene signature analysis (Supplementary Figure S1). False discovery rate (FDR) analysis using Benjamini and Hochberg Hochbergnat was used to count for multiple comparisons and tests (Supplementary Table S1). All tests are two-sided and p-values ≤ 0.05 were considered statistically significant.

## SUPPLEMENTARY MATERIALS FIGURES AND TABLES












