# Supplementary material for: Reciprocal expression of INSM1 and YAP1 defines subgroups in small cell lung cancer
Source: Oncotarget. 2017 Aug 28;8(43):73745–56. doi: 10.18632/oncotarget.20572 (PMC5650296; doi:10.18632/oncotarget.20572)
Supplement: Supplementary file 1 [file oncotarget-08-73745-s001.pdf]

Reciprocal expression of INSM1 and YAP1 defines subgroups in small cell lung cancer

SUPPLEMENTARY MATERIALS

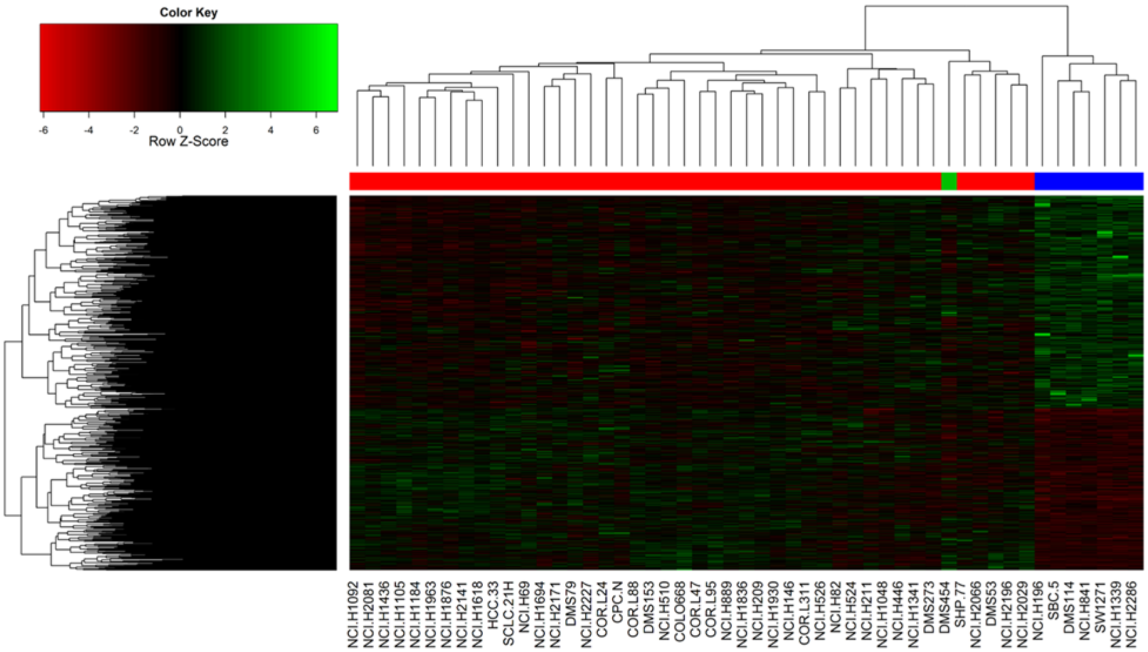

Supplement Figure S1. Gene expression clustering of SCLC cell lines. Names of cell lines given at bottom of heatmap. Colored bars at top indicate subgroup assignments: red = Group I, blue = Group II, and green = Group III. Data was downloaded from the Cancer Cell Line Encyclopedia (CCLE).

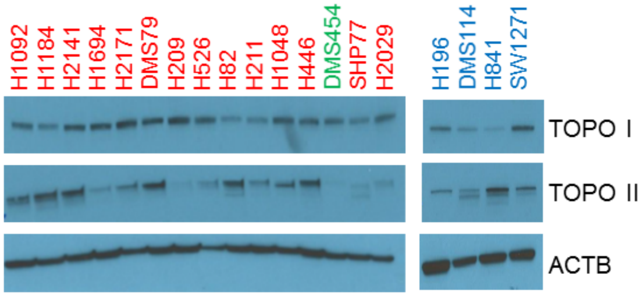

Supplement Figure S2. Topoisomerase I and II expression in SCLC cell lines. Western blots of protein lysates. Targeted protein listed on right. Cells are arranged on blot, left to right, in identical order as shown on clustering diagram in Figure 1A. Cell names are written in colored text to designate their subgroup classification. Group II cells were run on a separate gel but otherwise analyzed on the same day as Group I cells. β-actin (ACTB) was used as a loading control.

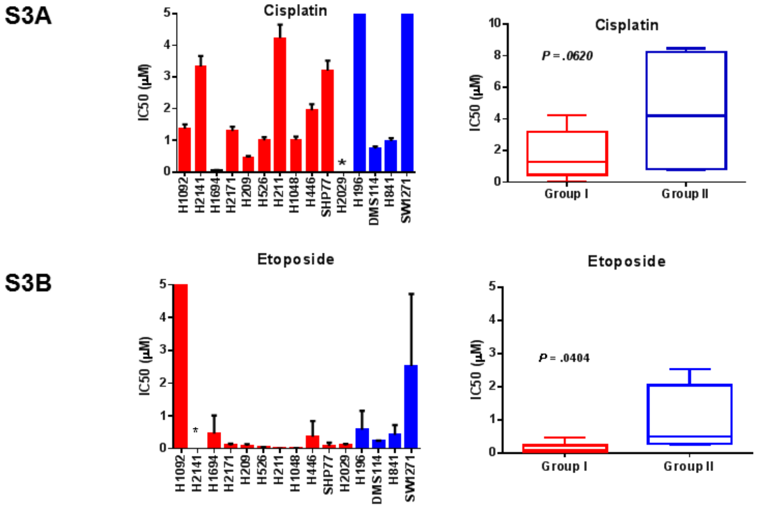

Supplement Figure S3. Chemo-sensitivity of SCLC subgroups. (A) IC50 values for cisplatin inhibition of cell growth listed for individual cell lines (left) as well as for cell line subgroups (right). Individual cell line IC50 values represent mean + SEM of 1-2 independent experiments and are shown as either red (Group I) or blue (Group II) bars depending on their subgroup assignment. Individual bars with no error bars have IC50 values > 5 μM. Cells are arranged on x-axis, left to right, in identical order to the clustering diagram in Figure 1A. *Cell line not tested. Boxplots represent mean ± SEM of individual IC50 values for a Group with *p* values showing significance between Groups. (B) Same as in panel A except for etoposide. The boxplot on right does not include H1092 data because it is an obvious outlier. The *p* value with H1092 data = 0.8792.

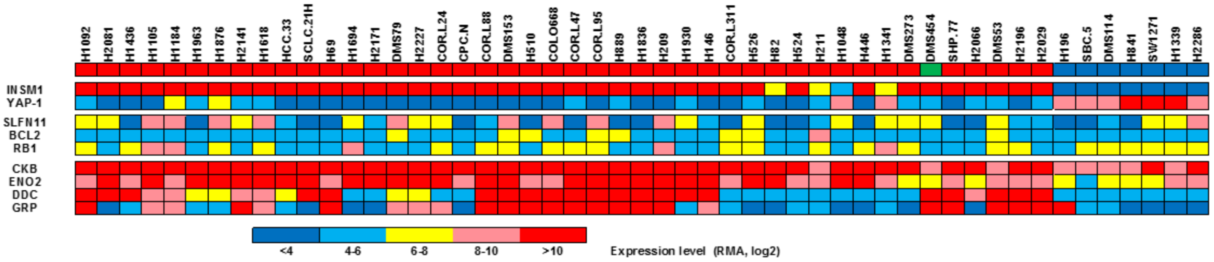

Supplement Figure S4. Expression of various genes in individual SCLC cell lines. Cell lines are arranged, left to right, following their gene expression clustering assignments, as shown in Figure 1A. Gene mRNA expression for individual genes, listed on left, was color-coded based upon the RMA, log2 values, obtained from the CCLE. Values for *SLFN11*, *BCL2*, and *RB1* are given for reference to their correlation with SCLC subgroups and drug sensitivity experiments. Values are given for creatine kinase isoform B (*CKB*), brain-specific enolase 2 (*ENOS2*), DOPA decarboxylase (*DDC*) and gastrin releasing peptide (*GRP*) because these are the four genes used by Gadzar and Minna for their original classification of SCLC cell lines (reference 7).

For Supplementary Tables see in Supplementary Files
