# Supplementary material for: Reciprocal expression of INSM1 and YAP1 defines subgroups in small cell lung cancer
Source: Oncotarget. 2017 Aug 28;8(43):73745–56. doi: 10.18632/oncotarget.20572 (PMC5650296; doi:10.18632/oncotarget.20572)
Supplement: Supplementary file 6 [file oncotarget-08-73745-s006.docx]

**Supplement Table S5: Descriptive statistics of SCLC cohorts.**

| **FACTOR** | **Discovery Cohort**  **FREQUENCY**  (*N* = 22) | **Second Cohort**  **FREQUENCY**  (*N* = 55) | **Genomic Cohort**  **FREQUENCY**  (*N* = 64) |
| --- | --- | --- | --- |
| **SEX**  Male  Female | 16  6 | 30  25 | 34  30 |
| **STAGE***  Extensive  Limited | 13  7 | 36  11 | 51  13 |

* Unknowns not recorded.
